# Subclinical Cardiac Disturbances After *Rickettsia* spp. Infection in an Endemic Region of Mexico

**DOI:** 10.3390/tropicalmed11030065

**Published:** 2026-02-26

**Authors:** Jeanny Fernanda Chapuz-Magaña, Nina Mendez-Dominguez, Karla Dzul-Rosado, Edgar Villarreal-Jimenez, Amonario Olivera-Mar, Vida Merry Salazar-Tostado, Miguel Santaularia-Tomas

**Affiliations:** 1 Hospital Regional de Alta Especialidad de la Peninsula de Yucatan, Servicios de Salud del Instituto Mexicano del Seguro Social para el Bienestar IMSS-BIENESTAR, Merida 97130, Mexico; ferchapuz1109@gmail.com (J.F.C.-M.); amar.hraepy@imssbienestar.gob.mx (A.O.-M.); vidatostado@uadec.edu.mx (V.M.S.-T.); 2 Centro de Investigaciones Regionales Dr. Hideyo Noguchi, Universidad Autonoma de Yucatan, Merida 97203, Mexico; karla.dzul@correo.uady.mx; 3 Health Sciences, Universidad Autonoma de Yucatan, Merida 97203, Mexico

**Keywords:** *Rickettsia*, subclinical cardiac involvement, Holter monitoring, follow-up study, endemic diseases

## Abstract

**Background:** Rickettsial diseases are endemic in southeastern Mexico, yet their potential subclinical cardiac effects remain poorly understood. Although severe spotted fever and typhus group infections may cause myocarditis and arrhythmias, limited evidence exists regarding cardiac alterations in individuals previously diagnosed with rickettsiosis who later show *Rickettsia* spp. IgG seropositivity. **Methods:** This follow-up observational study was conducted at a tertiary referral hospital in the Yucatan Peninsula. From an initial cohort of 390 patients evaluated for suspected rickettsial disease, 284 were confirmed as IgG-positive during follow-up. Among them, 18 adults who were asymptomatic for acute rickettsiosis at reassessment, but reported mild or nonspecific cardiac symptoms, underwent standardized cardiological evaluation. Procedures included a 12-lead electrocardiogram (ECG), transthoracic echocardiography, and 24 h Holter monitoring. All studies were reviewed independently by two blinded cardiologists with senior adjudication. **Results:** Global systolic function was preserved in all participants. However, subclinical abnormalities were identified, including right ventricular dilation in 16.7%, clinically relevant QTc prolongation in 22.2%, sinus pauses in 11.1%, reduced heart rate variability in 11.1%, atrial flutter in one patient, and complete left bundle branch block in one patient. QTc prolongation was detected exclusively through Holter monitoring. **Conclusions:** Adults previously diagnosed with rickettsiosis may exhibit subclinical cardiac involvement despite apparent recovery. Holter monitoring appears more sensitive than ECG for identifying electrical disturbances, warranting larger prospective studies.

## 1. Introduction

Rickettsial diseases, including Rocky Mountain spotted fever (RMSF) and Murine typhus (MT), are acute febrile illnesses caused by obligate intracellular bacteria of the genus *Rickettsia*. These infections are transmitted primarily by ticks, with *Rickettsia rickettsii* and *Rickettsia typhi* being the most pathogenic species affecting humans. In Mexico and other endemic regions, rickettsioses remain a significant public health problem due to delayed diagnosis, limited access to early serological testing, and overlapping clinical presentations with other acute febrile syndromes [1]. Although classic manifestations include fever, headache, rash, and systemic endothelial injury, multisystem involvement is common, particularly in severe or untreated cases.

Cardiovascular complications represent an underdiagnosed but clinically relevant component of rickettsial disease. Endothelial injury and subsequent vasculitis are the hallmarks of severe rickettsiosis and may affect the coronary microcirculation, conduction system, and myocardial tissue [2]. Case reports and small series have documented diverse cardiac manifestations, including atrial fibrillation [3], heart block and life-threatening arrhythmias [4], acute myocarditis with elevated cardiac enzymes and systolic dysfunction [5], and cardiac tamponade secondary to pericarditis [6]. Multimodality imaging studies further demonstrate that myocardial inflammation may occur even in previously healthy individuals and may initially present with preserved systolic function, detectable only through strain imaging or cardiac magnetic resonance (CMR) techniques [7]. Although most published cases describe severe presentations, emerging evidence suggests that subclinical myocardial involvement may be more common than recognized, particularly among individuals with recent *Rickettsia* exposure. However, systematic evaluation using electrocardiography, transthoracic echocardiography, and ambulatory rhythm monitoring remains rare outside isolated reports.

In the Yucatan Peninsula, recent clinical observations have documented the circulation of *Rickettsia rickettsii* and *Rickettsia typhi*, often presenting with nonspecific febrile syndromes easily confused with other tropical infections or post-COVID inflammatory conditions in children, underscoring their endemicity and diagnostic complexity [8]. Evidence from scrub typhus also highlights a broad spectrum of cardiac abnormalities, including tachyarrhythmias, bradyarrhythmias, systolic dysfunction, right ventricular involvement, and pericardial effusion, with probable myocarditis identified as an independent predictor of mortality [9].

Detailed imaging findings further support the link between *Rickettsia* infection and myocardial inflammation, showing myocardial edema, impaired regional strain, and late gadolinium enhancement on CMR, even with preserved ejection fraction; while isolated reports of arrhythmias, conduction disturbances, fulminant myocarditis, and cardiac tamponade in RMSF reinforce the pathogenic potential of these infections [7,10]. Despite this, systematic cardiac assessment after rickettsial disease is uncommon.

Given the combination of endemic circulation, possible cardiovascular involvement, and the absence of standardized cardiac screening, major knowledge gaps persist regarding early or silent cardiac abnormalities following *Rickettsia* spp. infection. Clarifying these subclinical findings is clinically relevant, as even mild myocardial injury, conduction disturbances, or pericardial involvement may predispose patients to future arrhythmias, ventricular dysfunction, or long-term sequelae.

## 2. Materials and Methods

### 2.1. Study Design and Setting

This study was designed as a follow-up observational investigation conducted at the Regional High Specialty Hospital of the Yucatan Peninsula (HRAEPY), an IMSS-Bienestar tertiary referral center located in Mérida, Mexico. The hospital serves a population residing in an endemic region for rickettsial diseases, where *Rickettsia rickettsii* and *Rickettsia typhi* circulate regularly.

Patients were initially evaluated in the hospital for suspected rickettsial infection as part of routine clinical care. During this first encounter, diagnostic testing was performed, and individuals with positive serology for *Rickettsia* spp. were documented and subsequently followed. As part of this follow-up process, patients who became IgG-positive were recontacted and invited to undergo cardiological assessment approximately 5 months after the serology test, once the acute phase had resolved.

The follow-up evaluation included adults who were asymptomatic for acute rickettsiosis at the time of reassessment, but who reported mild or nonspecific cardiac symptoms or were clinically eligible for cardiac evaluation. Cardiological assessments were performed at the same institution and included resting 12-lead electrocardiography (ECG), two-dimensional transthoracic echocardiography, and 24 h Holter monitoring. All diagnostic studies were conducted using standardized institutional protocols by certified technicians and interpreted independently by two blinded cardiologists, with senior adjudication in cases of disagreement.

This design allowed the identification of potential subclinical or delayed cardiac abnormalities following confirmed *Rickettsia* spp. infection, capturing cardiac status after serological evidence of exposure rather than during acute illness.

### 2.2. Participants

A total of 390 patients were initially evaluated at HRAEPY for suspected rickettsial disease as part of routine clinical care. During this first clinical encounter, diagnostic testing was performed, and *Rickettsia* spp. serology (IgG) was obtained. Of these, 284 individuals were confirmed as IgG-positive by IFA and were followed up. From this IgG-positive cohort, adults aged ≥18 years were considered eligible for cardiological reassessment.

Inclusion criteria: Documented *Rickettsia* spp. IgG positivity during follow-up; absence of acute rickettsial symptoms (Fever, rash and headache) at the time of reassessment (asymptomatic for rickettsiosis); negative result in test for other endemic infectious diseases including Chagas; presence of mild or nonspecific cardiac symptoms (e.g., palpitations, dyspnea, transient dizziness) or clinical indication for cardiological evaluation; ability and willingness to complete all cardiology studies (ECG, echocardiogram, and Holter monitoring); provision of written informed consent.

Exclusion criteria: Acute symptomatic rickettsiosis requiring treatment or hospitalization at the time of reassessment or last known rickettsial infection; known pre-existing structural heart disease; documented arrhythmias, cardiomyopathy, or significant valvular disease unrelated to rickettsial infection; incomplete cardiologic evaluation or poor-quality imaging/monitor tracings preventing interpretation. A total of 18 patients met eligibility criteria and completed the full cardiological evaluation.

### 2.3. Serology Test

To determinate the seropositivity of *Rickettsia*, IgG antibody titters were determined by indirect immunofluorescence assay (IFA).

Human sera were examined via IFA protocol using crude antigens from *Rickettsia rickettsia* [11] and *Rickettsia typhi* [12] slides prepared in-house. The IFA was performed according to the methodology described before for Dzul-Rosado et al. [13] A cutoff dilution of 1:64 was used to determine the seropositivity result.

### 2.4. Procedures

All eligible participants underwent a standardized cardiological evaluation during follow-up, after the resolution of acute rickettsial symptoms and confirmation of *Rickettsia* spp. IgG seropositivity. The evaluation consisted of three diagnostic procedures performed on the same day:

1. A standard 12-lead resting electrocardiogram was obtained using hospital digital equipment. Recorded variables included heart rate, rhythm, PR interval, QRS duration, QT/QTc interval, frontal plane axis, conduction patterns, and ST–T wave morphology. Measurements and interpretations followed European Society of Cardiology (ESC) reference criteria and international guidelines.
2. A two-dimensional transthoracic echocardiogram was performed in accordance with recommendations from the American Society of Echocardiography and the ESC. Parameters assessed included:
  ○ Left ventricular ejection fraction (LVEF)
  ○ Chamber dimensions and volumes
  ○ Regional wall motion
  ○ Diastolic function
  ○ Right ventricular performance (including TAPSE and strain when available)
  ○ Pericardial assessment
  ○ Estimated pulmonary artery systolic pressure (PASP)

Participants underwent continuous 24 h ambulatory rhythm monitoring using a three-channel Holter device.

- Supraventricular and ventricular ectopic burden
- Bradyarrhythmias and tachyarrhythmias
- Sinus pauses
- Heart rate variability
- QTc measurement (automatic and manual)
- ST-segment deviations suggestive of ischemia or inflammation

Clinically significant QTc prolongation was defined using sex-specific thresholds (>450 ms in men and >460 ms in women), based on manual cardiologist adjudication of Holter recordings. Fridericia correction was preferentially considered in the presence of marked heart rate variability, while automated Bazett-corrected values were used only as supportive data.

ECGs, echocardiograms, and Holter studies were independently evaluated by two experienced cardiologists who were blinded to clinical history and serological data. In cases of disagreement, a senior cardiology professor adjudicated the final interpretation to ensure diagnostic consistency and minimize observer bias.

### 2.5. Variables and Data Collection

Collected variables included age and sex; relevant comorbidities (e.g., hypertension, diabetes, dyslipidemia, obesity); Cardiovascular risk factors; history of suspected or confirmed rickettsial disease; symptom status at follow-up, with specific documentation of mild or nonspecific cardiac symptoms (e.g., palpitations, dyspnea, dizziness)

Serological testing included *Rickettsia* spp. IgG positivity (Spotted Fever Group or Typhus Group), serological results were used only to confirm prior infection; no serology was performed on the day of cardiac evaluation.

Cardiac measurements were obtained from ECG, echocardiography, and Holter monitoring. Extracted variables included:

- Heart rate and rhythm
- PR, QRS, and QT/QTc intervals
- Axis deviation
- Conduction abnormalities
- ST–T wave morphology
- Left ventricular ejection fraction
- Global longitudinal strain (GLS) when available
- Right ventricular function (including TAPSE)
- Chamber size and wall motion
- Diastolic function
- Estimated pulmonary artery systolic pressure
- Pericardial findings
- Minimum, mean, and maximum heart rate
- Supraventricular and ventricular ectopic frequency
- Sinus pauses
- Tachyarrhythmias and bradyarrhythmias
- Heart rate variability
- QTc intervals (automatic and manual)
- ST-segment deviations suggestive of ischemia or inflammation

Data were reviewed independently by two blinded cardiologists, with adjudication by a senior cardiology specialist when discrepancies occurred.

### 2.6. Statistical Analysis

Given the exploratory nature of this follow-up study and the small sample size, analyses were limited to descriptive statistics. Continuous variables were summarized using means and standard deviations or medians and interquartile ranges (IQR), depending on distribution. Categorical variables were reported as frequencies and percentages; confidence intervals were not calculated given the exploratory nature of the study and the very small sample size, which would yield extremely wide and clinically uninformative estimates.

No inferential hypothesis testing was performed, as the study was not powered to detect statistical differences and did not include a comparison group. Instead, emphasis was placed on describing the frequency and characteristics of cardiologic abnormalities identified through ECG, echocardiography, and Holter monitoring.

For variables with clinical thresholds, classification followed established international cardiology guidelines. When appropriate, proportions were accompanied by descriptive context to illustrate the magnitude of findings in relation to typical population expectations.

### 2.7. Ethical Considerations

This study was conducted in accordance with the ethical principles outlined in the Declaration of Helsinki and all applicable national research regulations. The protocol was reviewed and approved by the Institutional Research and Ethics Committee of the Regional High Specialty Hospital of the Yucatan Peninsula (HRAEPY), IMSS-Bienestar (approval number: 2024-033; April 2025).

All participants provided written informed consent prior to enrollment in the follow-up cardiological evaluation. Consent procedures included explanation of the study purpose, voluntary participation, confidentiality safeguards, and the right to withdraw at any time without impact on clinical care. Patient privacy and data confidentiality were strictly protected. All clinical and cardiological information was collected and stored using institutional secure systems accessible only to authorized research personnel. Because this investigation involved only follow-up evaluation and non-invasive cardiological procedures, it was classified as minimal-risk observational research by the institutional committee.

## 3. Results

### 3.1. Sociodemographic and Clinical Characteristics

A total of 390 patients were screened for suspected rickettsiosis. Of these, 284 (72.8%) tested positive for *Rickettsia* spp. by IgG serology. Among the seropositive individuals, 18 met the inclusion criteria and completed the full cardiological evaluation, which consisted of electrocardiography, transthoracic echocardiography, and 24 h Holter monitoring. Sociodemographic characteristics and the patient flow process are summarized in Table 1.

During directed clinical interviews, 11 patients reported nonspecific symptoms, while seven described cardiovascular-related manifestations such as dyspnea, peripheral edema, tachycardia, and cyanosis. Regarding classic symptoms of rickettsiosis, one patient reported fever > 38 °C, three noted photophobia, four experienced myalgia, and two described dermatologic findings, including macular or papular erythema. Several of these symptoms were difficult to attribute exclusively to rickettsial infection because of concomitant comorbidities.

The identified *Rickettsia* species were classified according to the four recognized groups of the genus. In this cohort, 13 patients (72.2%) were infected with species of the Spotted Fever Group, four (22.2%) were classified under the Typhus Group, and in one patient (5.6%) the specific *Rickettsia* species could not be determined. The predominance of Spotted Fever Group infections is consistent with the known epidemiological profile of the region.

### 3.2. Cardiological Findings

#### 3.2.1. Echocardiographic Findings

Echocardiographic parameters were largely within normal limits for the cohort. Mean values demonstrated preserved systolic function, including a left ventricular ejection fraction of 63.1%, TAPSE of 24.1%, and global longitudinal strain of –21.8%, all of which fall within normal reference ranges. No significant valvular gradient elevations were observed and estimated pulmonary systolic pressures remained below thresholds suggestive of pulmonary hypertension (PSAP < 25 mmHg).

Age-expected patterns of diastolic function were observed without evidence of restrictive physiology or diastolic dysfunction out of proportion to normal aging. However, three patients (16.7%) demonstrated right ventricular dilation, a finding that may signal subclinical cardiac involvement potentially associated with *Rickettsia* infection. These abnormalities warrant further investigation in prospective studies.

#### 3.2.2. Electrocardiographic Findings

Mean electrocardiographic values were within expected normal ranges for age and sex. No consistent abnormalities in conduction intervals, ST-segment morphology, or T-wave patterns were identified across the group. Cardiac axes remained normalized.

Two clinically relevant findings were recorded, atrial flutter in one patient, later confirmed by Holter monitoring and complete left bundle branch block (LBBB) in one patient, with no prior documented history. These two patients were identified as requiring cardiology follow-up and potential treatment based on electrocardiographic criteria. The remaining patients did not require immediate cardiovascular intervention but were advised to maintain clinical surveillance, especially if symptoms progress or new manifestations occur.

#### 3.2.3. Holter Monitoring Findings

Holter monitoring revealed a mean heart rate of 76.3 bpm, with minimum and maximum values of 46.8 bpm and 132.8 bpm, respectively, indicating preserved chronotropic competence. More than 99% of beats corresponded to sinus rhythm, confirming overall electrical stability in most patients.

There was a notable discrepancy between automated and manual QTc measurements:

- Automated Holter QTc means: 501.5 ms
- QT using Fridericia correction: 417.7 ms, by an electrophysiology cardiologist

The overestimation by automated algorithms based on Bazzet, attributed to artifact and heart rate variability. Based on manual interpretation, 4/18 (22.2%) had QTc prolongation, 3/18 had right ventricular dilation.

Isolated electrical abnormalities included: (a) Pauses > 2 s in two patients (2.1 s and 2.4 s); (b) Reduced heart rate variability in two patients (c) Atrial flutter in one patient (d) Complete LBBB in one patient (e) Significant ectopic burden in three patients (supraventricular and/or ventricular).

No sustained tachyarrhythmias, high-grade ventricular ectopy, or life-threatening arrhythmias were identified, overall classification is presented in Table 2. Collectively, Holter findings suggest subclinical electrical disturbances in a subset of patients, possibly reflecting endothelial or inflammatory consequences of prior *Rickettsia* exposure.

## 4. Discussion

In the present study, cardiac abnormalities were identified in a group of patients with IgG-seropositive *Rickettsia* spp. infection, suggesting the presence of subclinical cardiac alterations detectable only through systematic cardiological evaluation. Although conventional echocardiographic parameters (including LVEF, TAPSE, and global longitudinal strain) remained within normal limits for most participants, subtle findings such as right ventricular dilation and electrical disturbances detected by Holter monitoring indicate that the cardiac effects of prior rickettsial exposure may extend beyond overt clinical disease. These observations are consistent with the pathophysiology of rickettsial infections, which target endothelial cells and can induce localized vasculitis, microvascular dysfunction, and autonomic alterations even in the absence of severe systemic manifestations [14].

One of the most relevant findings was the discrepancy between resting ECG and Holter measurements, particularly regarding QTc prolongation. While 12-lead ECGs were largely normal, Holter monitoring identified QTc prolongation in 4 of 18 patients. Similar findings have been reported in other vector-borne diseases, such as scrub typhus, where QTc prolongation has been described in approximately 19% of cases and has been attributed, at least in part, to infection-related inflammatory responses [15,16].

This discrepancy likely reflects fundamental differences between the two modalities: ECG provides a brief snapshot of cardiac electrical activity, whereas Holter monitoring captures 24 h physiological variability, including autonomic fluctuations and transient inflammatory or microvascular effects. Automated Holter algorithms may overestimate QTc in the presence of artifacts or heart rate variability, reinforcing the importance of expert manual electrophysiological interpretation. Prior studies support the superior specificity of Holter monitoring for detecting clinically relevant electrical abnormalities compared with resting ECG alone [17], and evidence indicates that Fridericia correction offers more accurate QT adjustment than Bazett during ambulatory monitoring [18].

The pattern of QTc prolongation and isolated arrhythmic findings observed in this cohort, including atrial flutter and conduction abnormalities, aligns with growing international evidence of myocardial involvement in spotted fever and typhus group rickettsioses. Supraventricular arrhythmias have been reported in association with rickettsial infections and may resolve with timely antimicrobial therapy [3,19]. Other studies describe transient conduction delays and ventricular irritability linked to inflammatory or autonomic mechanisms triggered by rickettsial vasculitis [4]. Endothelial injury has also been associated with coronary ectasia, transient coronary obstruction, and microvascular ischemia, highlighting vascular inflammation as a central mechanism underlying cardiac manifestations [20,21]. The role of cyclooxygenase-2 expression in endothelial inflammation further supports these mechanisms [22]. This spectrum parallels findings in other vector-borne bacterial diseases, particularly Lyme disease, where immune-mediated inflammation affects the myocardium and conduction system [23].

Although the cohort was small, the observed abnormalities may represent the milder end of a continuum ranging from asymptomatic endothelial activation to fulminant myocarditis. Preserved systolic function is reassuring; however, the detection of QTc prolongation, conduction delays, reduced heart rate variability, and sinus pauses suggests potential persistent myocardial irritability following Rickettsia spp. exposure. Notably, these abnormalities were detected in a population largely asymptomatic at follow-up, underscoring the risk of underdiagnosis in endemic regions.

These findings are consistent with epidemiological patterns in Yucatán, where variations in spotted fever and typhus group distribution have been reported [24], and where *Rickettsia rickettsii* and *Rickettsia typhi* increasingly contribute to systemic inflammatory presentations that are frequently misdiagnosed as other tropical infections or SARS-CoV-2–related syndromes [8,14]. This diagnostic overlap may delay recognition and limit opportunities for appropriate cardiac surveillance. In this context, our results support the integration of Holter monitoring and transthoracic echocardiography into follow-up evaluations of patients with confirmed or suspected rickettsial infection.

This study has several limitations. The small sample size and absence of a seronegative control group limited statistical power and precluded inferential analyses. Although strict inclusion criteria and standardized assessments were applied to enhance internal validity, the observational design limits conclusions regarding temporal relationships between infection and cardiac findings. In addition, while echocardiography and Holter monitoring provide valuable functional and electrical information, subtle myocardial inflammation or fibrosis may require advanced imaging such as cardiac MRI. Reliance on IgG serology does not allow differentiation between single and repeated exposures, and participants who completed follow-up may represent a subgroup with greater symptom awareness or health-seeking behavior, potentially overestimating the frequency of abnormalities. Finally, nonspecific symptoms such as dyspnea or palpitations may be influenced by comorbid conditions, although emphasis on objective cardiologic markers helped reduce misclassification.

Despite these limitations, this study provides novel evidence of subclinical cardiac involvement following *Rickettsia* spp. infection and highlights the utility of Holter monitoring as a diagnostic tool in endemic regions.

## 5. Conclusions

Our results suggest that patients with previous *Rickettsia* spp. infection who were asymptomatic for acute rickettsiosis could demonstrate electrical abnormalities, despite largely preserved systolic function on echocardiography. The identification of rhythm disturbances aligns with isolated cases reported in the literature and reinforces that rickettsial infections may exert cardiac effects even in the absence of overt clinical symptoms. QTc prolongation was detected in 22.2% (4/18) of participants, highlighting the value of systematic Holter monitoring, which proved more sensitive than resting ECG for detecting subtle electrical alterations influenced by autonomic variability, microvascular inflammation, or endothelial dysfunction.

Given the endemicity of rickettsial diseases in the region and their known capacity to cause endothelial injury and conduction abnormalities, our findings suggest that individuals with confirmed or suspected exposure, particularly those with cardiovascular comorbidities, may benefit from structured cardiological evaluation, including ambulatory rhythm monitoring.

Overall, this exploratory study indicates that subclinical cardiac involvement may occur in individuals with past *Rickettsia* spp. exposure. However, it is necessary to conduct more research with a large sample size to identify the frequency and factors that may influence the general population. Prospective investigations with larger cohorts and longitudinal follow-up are needed to determine the prognostic significance of these abnormalities, clarify underlying mechanisms, and inform evidence-based recommendations for cardiac surveillance in endemic populations.

## Figures and Tables

**Table 1:** Sociodemographic Characteristics of the Evaluated Patients (number = 18).

Characteristic	Value
Age, median (IQR)	54.5 (27)
Male, n (%)	5 (28)
Female, n (%)	13 (72)
BMI, median (IQR)	30.4 (9.1)
Normal, n (%)	2 (11)
Overweight n (%)	4 (22)
Obesity n (%)	7 (39)
Missing (%)	5 (28)
Heart rate, median (IQR)	75 (17)
Diabetes mellitus, n (%)	3 (17)
Hypertension, n (%)	4 (22)
Dyslipidemia, n (%)	4 (22)

**Table 2:** Cardiac Abnormalities Detected Across Modalities in patients diagnosed with rickettsiosis (n = 18).

Echocardiography		
Parameter	Mean ± SD/Findings	Interpretation
LVEF (%)	63.10%	Normal systolic function
TAPSE (mm)	24.1 mm	Normal RV systolic function
Global longitudinal strain (%)	–21.8%	Normal strain
Pulmonary systolic pressure (PSAP)	<25 mmHg	No pulmonary hypertension
Valvular gradients	Normal	No significant valvular disease
Diastolic function	Age-expected	No pathological restriction
Right ventricular dilation	3 patients (16.7%)	Possible mild involvement
Electrocardiography (Resting 12-lead ECG)		
ECG Variable/Finding		Result
Mean heart rate	68.1 bpm	Within normal range
Rhythm		Mostly sinus rhythm
Conduction intervals (PR, QRS, QT)149.6 ms, 92.3 ms, 390 ms		Normal in most patients
Axis	25.1°	Normal
ST–T abnormalities		Unremarkable
Notable findings		
Atrial flutter		1 patient
Complete left bundle branch block (LBBB)		1 patient
Clinical relevance		2 patients in surveillance
Holter Monitor (24 h)		
Parameter	Mean/n (%)	Notes
Mean heart rate (bpm)	76.3 (range 46.8–132.8)	Preserved chronotropic response
% sinus beats	>99%	Indicates electrical stability
Automatic QTc (mean)	501.5 ms	Overestimated due to artifacts
Manual QTc (mean)	417.7 ms	Electrophysiologist-reviewed
QTc prolongation	4 patients (22.2%)	Clinically relevant
Isolated findings		
Sinus pauses > 2 s	2 patients (2.1 and 2.4 s)	Autonomic dysfunction possible
Reduced HR variability	2 patients	Suggests autonomic involvement
Atrial flutter	1 patient	Confirmed on ECG
Complete LBBB	1 patient	Same as ECG
Significant ectopy	3 patients	Mild myocardial irritability
Serious arrhythmias	None	No sustained VT/VF
Summary Across Modalities		
Modality	Finding	n (%)
Echocardiogram	Right ventricular dilation	3 (16.7%)
ECG	Atrial flutter	1 (5.6%)
ECG	LBBB	1 (5.6%)
Holter	QTc prolongation	4 (22.2%)
Holter	Sinus pauses	2 (11.1%)
Holter	Reduced HRV	2 (11.1%)
Holter	Significant ectopy	3 (16.7%)

## Data Availability

Dataset available on request from the corresponding authors.

## Abbreviations

The following abbreviations are used in this manuscript:

RMSF	Rocky Mountain Spotted Fever
MSF	Mediterranean Spotted Fever
CMR	Cardiac Magnetic Resonance
ECG	Electrocardiogram
ESC	European Society of Cardiology
LBBB	Left Bundle Branch Block
